# Critical Role of Tumor Necrosis Factor Signaling in Mesenchymal Stem Cell-Based Therapy for Autoimmune and Inflammatory Diseases

**DOI:** 10.3389/fimmu.2018.01658

**Published:** 2018-07-20

**Authors:** Li Yan, Dejin Zheng, Ren-He Xu

**Affiliations:** Faculty of Health Sciences, University of Macau, Taipa, Macau, China

**Keywords:** mesenchymal stem cells, tumor necrosis factor, TNFR, regulatory T, autoimmune and inflammatory diseases

## Abstract

Mesenchymal stem cells (MSCs) have been broadly used as a therapy for autoimmune disease in both animal models and clinical trials. MSCs inhibit T effector cells and many other immune cells, while activating regulatory T cells, thus reducing the production of pro-inflammatory cytokines, including tumor necrosis factor (TNF), and repressing inflammation. TNF can modify the MSC effects *via* two TNF receptors, i.e., TNFR1 in general mediates pro-inflammatory effects and TNFR2 mediates anti-inflammatory effects. In the central nervous system, TNF signaling plays a dual role, which enhances inflammation *via* TNFR1 on immune cells while providing cytoprotection *via* TNFR2 on neural cells. In addition, the soluble form of TNFR1 and membrane-bound TNF also participate in the regulation to fine-tune the functions of target cells. Other factors that impact TNF signaling and MSC functions include the gender of the host, disease course, cytokine concentrations, and the length of treatment time. This review will introduce the fascinating progress in this aspect of research and discuss remaining questions and future perspectives.

## Introduction

Among many multipotent stem cell types, mesenchymal stem cells (MSCs) are a unique cell type that possesses not only stem cell properties but also immunomodulatory capabilities. MSCs refer to multipotent cells derived from the mesenchyme—the embryonic connective tissue that originates from the mesoderm. MSCs can differentiate into a wide variety of cells from the mesoderm, including osteocytes, chondrocytes, adipocytes, and smooth muscle cells (1, 2), and some cell types from the other germ layers, such as neurons from the ectoderm (3, 4) and hepatocytes from the endoderm (5, 6). Recently, neural crest cells were identified as another source giving rise to mesenchymal progenitors, which, similar to MSCs, have a high potential to differentiate into osteocytes and chondrocytes (7, 8). MSCs can be isolated from many fetal and adult tissues or differentiated from human pluripotent stem cells (hPSCs). *In vitro* and *in vivo* studies have demonstrated that MSCs modulate immune responses and inflammation and execute cytoprotective and reparative effects mainly through cell–cell contact and paracrine mechanisms. Thus, MSCs have been used as a cell therapy for an increasing number of autoimmune, inflammatory, and degenerative diseases (1, 2).

Autoimmunity and chronic inflammation are known to share numerous factors, and thus, frequently coexist in the same patients. Autoimmune disease occurs when the immune system abnormally attacks a part of a normal body. Approximately 80 types of autoimmune diseases have been identified, and these diseases can involve almost any part of the body. The abnormal immune response is often associated with complicated genetic factors and the environment. Autoimmune disease is a common and often serious clinical problem due to the chronic nature, high incidence in human populations, especially in women, and rising cost of healthcare. Among the list of common autoimmune diseases, rheumatoid arthritis (RA) (9), inflammatory bowel disease (IBD) (10), and type-1 diabetes (T1D) (11) are on the top. Approximately 7% of people in the United States are affected by autoimmune disease. Tumor necrosis factor (TNF or TNFα), which is involved in a wide range of biological functions, is considered the master mediator of the pathogenesis of chronic inflammation and autoimmune diseases. Therefore, anti-TNF therapies have become mainstay treatments for autoimmune and inflammatory diseases.

Mesenchymal stem cells are susceptible to environmental changes, and their immunosuppressive functions can be modulated when exposed to an inflammatory milieu (12). TNF and other pro-inflammatory cytokines, such as interferon γ (IFNγ) and interleukin 1 (IL-1), determine the disease onset, severity, and relapse of autoimmune diseases and affect the efficacy of treatment, including MSC-based therapy. IFNγ, TNF, and IL-1 present in inflammatory tissues can augment the immunosuppressive functions of MSCs (13–15). Priming of MSCs with IFNγ can yield an augmented immunosuppressive population with a higher efficacy for anti-inflammatory treatment than non-primed MSCs (16). Primed MSCs have been broadly applied in both basic and clinical research (17). However, no focused review has discussed the role of TNF signaling in MSC-based therapy of autoimmune and inflammatory diseases, given the great progress in this area of research. TNF exerts its functions by binding to two receptors (TNFR1 and TNFR2) to regulate the survival, proliferation, migration, and differentiation of target cells, especially immune cells. This molecule also interacts with MSCs to modify or mediate their therapeutic effects. This review, aimed to introduce the progress in this area, will specifically discuss how TNF/TNFR and MSCs converge on the immune system to prevent autoimmune and inflammatory diseases.

## MSC Efficacy on Autoimmune and Inflammatory Diseases

Mesenchymal stem cells have tremendous potential as a cellular therapy for autoimmune and inflammatory diseases because of their strong immunomodulatory effects and tissue regenerative capability. A growing number of translational studies have been carried out on MSCs for the treatment of many autoimmune and inflammatory diseases, including T1D (18), RA (19), IBD (20), ulcerative colitis (21), systemic lupus erythematosus (SLE) (22), autoimmune uveitis (23, 24), and Sjogren’s syndrome (25). So far, over 5,000 MSC-related clinical trials have been registered at ClinicalTrials of the National Institutes of Health in the U.S. (https://clinicaltrials.gov/), of which over 1,900 trials have been completed. Both autologous and allogenic MSCs were used in these trials, in which bone marrow (BM), adipose tissue, umbilical cord, placenta, and dental pulp were the most common sources for MSCs. In addition, MSCs differentiated from hPSCs, including embryonic stem cells and induced pluripotent stem cells (iPSCs), have also been examined and demonstrated efficacy on a variety of animal disease models and may become new options for future clinical applications (21, 26–28).

Mesenchymal stem cells regulate the adaptive immune system by promoting the generation of regulatory T cells (Tregs) and repressing the functions of T effector (Teff) and B effector cells (29–31). These effects are mainly triggered by exposure to pro-inflammatory cytokines, such as TNF, IFNγ, and IL-1β, which are widely present in tissues affected by inflammatory and autoimmune diseases. For instance, TNF deregulates the balance between Tregs and pathogenic Th17 and Th1 cells in the synovium of RA patients and impairs Treg functions in RA and MS patients (32, 33). Systemically transplanting MSCs into patients leads to a decrease in the number of Teff cells and restoration of Treg functions (22, 34). Moreover, IFNγ-primed MSCs inhibit B cell differentiation by arresting the cell cycle and inducing apoptosis (35).

As for innate immunity, MSCs can inhibit natural killer (NK) cell cytotoxicity and block the differentiation and/or maturation of macrophages and dendritic cells (DCs). MSCs skew the polarization of macrophages from M1 to M2 in wound healing (36) and inhibit DC generation and migration to lymph nodes *in vivo* (37). Studies of the molecular mechanisms for the therapeutic effects of MSCs have revealed that MSCs modulate immune responses and promote tissue repair *via* secretion of soluble factors and direct cell–cell contact (29). MSCs exert immunosuppressive effect by secreting soluble factors, such as indoleamine 2,3-dioxygenase (IDO), prostaglandin E2 (PGE2), hepatocyte growth factor (HGF), transforming growth factor-β1, insulin-like growth factor-1 (IGF-1), nitric oxide, and human leukocyte antigen-G5 (38, 39). Inhibition of IDO or PGE2 synthesis results in reduction of MSC-mediated immunosuppression, and priming MSCs with pro-inflammatory cytokines, such as IFNγ and TNF, enhances the immunosuppressive effects by elevating the secretion of IDO, CXCR4, and PGE2 (29, 39–42). MSCs mixed with activated T cells have the strongest inhibition on the T cells *via* direct cell contact (43), and upregulated expression of intercellular adhesion molecule-1 and vascular adhesion molecule-1 in MSCs strengthens their interaction with T cells (44).

Although promising results have been obtained from MSC-based therapy, the outcomes are not always consistent and sometimes even contradictory, depending on the delivery strategies, MSC sources, and disease course (45–49). A phase I study reported that 7/10 patients with Crohn’s disease did not respond to autologous BM-MSC infusion, and three of them even manifested worsened symptoms (50). Site-specific administration of MSCs to patients with Crohn’s disease and mice with collagen-induced arthritis (CIA) appeared to be more effective than systemic injection (51, 52). It has been well documented that the functions of MSCs depend on the microenvironment. MSCs often manifest immunosuppressive effects in a strong inflammatory milieu, and this ability is reduced or lost and the immunogenicity of the cells increased in a weak inflammatory environment (2). Long-term exposure to IFNγ or TNF even converts MSCs from an immunosuppressive to pro-inflammatory status (53–55). Moreover, MSCs are effective at disease onset or when the symptoms reach peaks but fail to alleviate the symptoms after the disease stabilizes or during disease progression (46, 56).

In addition, the origin of MSCs also influences their immunomodulatory effects. For example, autologous BM-MSCs from patients with SLE or synovial-derived MSCs from patients with RA failed to improve the symptoms of the same donor patients (47, 57). Adipose-derived MSCs from mice with experimental autoimmune encephalomyelitis (EAE) had no therapeutic effect on the donor animals (58). MSCs isolated from obese mice or non-obese diabetic mice failed to alleviate the symptoms in EAE and T1D mice (18, 59). Thus, choosing MSCs from the right source and determining the immunomodulatory effects of MSCs are necessary before therapeutic applications.

## TNF Signaling

Currently, 19 members have been identified in the TNF superfamily (TNFSF), including TNF, TNFβ, CD40L, FasL, and TRAIL, which participate in diverse cellular activities, including inflammation, cell proliferation, apoptosis, and morphogenesis (60). In particular, TNF is abundant in the serum and many other body fluids in patients with autoimmune disease. TNF is a trimeric type-II transmembrane protein that shares a TNF homology domain with the other TNFSF members and is produced mainly by activated macrophages, T, B, and NK cells. TNF is present in two different forms, the membrane-bound TNF (mTNF) and soluble TNF (sTNF or TNF), and TNF is cleaved from mTNF *via* metalloproteinases, such as TNF-converting enzyme (TACE) (61–63).

Tumor necrosis factor and sTNF bind to two structurally distinct transmembrane receptors, TNFR1 and TNFR2, both belonging to the TNFR superfamily, which comprises trimeric type-I transmembrane proteins with repeated extracellular cysteine-rich domains for ligand binding; the two receptors regulate gene expression *via* different signaling pathways (61). TNFR1 can be activated by both mTNF and TNF, whereas TNFR2 preferentially binds to mTNF to initiate the activation of the receptor (64). Moreover, TNFR1 is expressed on almost all cells of the body, whereas TNFR2 is expressed only on limited cells, e.g., immune cells, endothelial cells, nerve cells, and MSCs. TNFR also includes membrane-bound (mTNFR or TNFR) and soluble (sTNFR) forms, and sTNFR is cleaved from TNFR by TACE (63).

In general, TNF induces cell apoptosis or survival through at least five different signals, including caspase, NFκB, ERK, JNK, and P38 MAPK pathways, *via* TNFR1 and -R2 (60). TNFR1 contains 434 amino acids, and its intracellular region contains a death domain (DD), which recruits the TNF-associated death domain (TRADD), and the latter then recruits Fas-associated death domain to trigger the caspase cascades and apoptosis. In addition, TNFR1 also induces reactive oxygen species release from mitochondria to activate apoptotic events. Paradoxically, TRADD can also recruit the TNFR-associated factor (TRAF2) to initiate the NFκB, ERK, JNK, and p38 MAPK signaling pathways to regulate the cell survival and proliferation. By contrast, TNFR2 consists of 439 amino acids and does not include a cytoplasmic DD, which binds to TRAF2 directly and activates pro-survival genes through the NFκB, ERK, JNK, and p38 MAPK pathways (60). There is some degree of cross talk between the TNFR1 and -R2 signaling pathways.

Another key feature of TNF signaling is the phenomenon called “reverse signaling,” in which the signal transmits from the TNFRs (including their membrane-bound and soluble forms) to mTNF-bearing cells (outside to inside). Reverse signaling of TNF has been shown to be functional in macrophages and T, B, and NK cells in humans. For example, activation of mTNF reverse signaling enhances the cytotoxicity of CD8 T cells and NK cells and the survival of B cells (65–67). In addition, soluble TNFR1 (sTNFR1)-stimulated monocytes manifest pro-inflammatory effects without TNF treatment and anti-inflammatory effects after TNF treatment, as reflected by regulation of the pro-inflammatory cytokines IL1β and IL8 (68). Moreover, the mTNF reverse signaling renders macrophage resistant to LPS-induced effects by inducing *TGFβ* expression (69, 70). It has been shown that the cytoplasmic domain of mTNF contains a casein consensus sequence, which is dephosphorylated during activation of the mTNF reverse signal. mTNF then triggers the p38 MAPK and JNK pathways *via* interaction with protein kinases. Alternatively, a 10-kDa cytoplasmic domain of mTNF can be cleaved and translocated into the nucleus to regulate the expression of various cytokines, such as *IL1β* and *IL12* (71). However, how the mTNF reverse signal works has not yet been fully understood.

## TNF in Autoimmune and Inflammatory Diseases

The important role of TNF in autoimmune and inflammatory disease has been supported by large amounts of evidence from clinical studies. TNF and sTNFR1 are recognized as useful indicators for assessing disease activity. For example, they are often at high levels in patients with RA and ankylosing spondylitis (72). In SLE patients, TNF is also elevated, and circulating sTNFR is significantly higher than in patients with RA and spondyloarthropathies (73). Chronic progressive MS patients manifest elevated TNF in CSF and active lesions compared with serum (74). The TNF level correlates with the manifestation and degree of disability in patients.

A vast number of animal studies have uncovered much more knowledge than clinical trials about the pathogenesis mediated by TNF. Transgenic mice overproducing TNF develop severe inflammatory arthritis, and the disease onset depends on IL1 production (75). IL17 promotes osteoclastogenesis by stimulating TNF production (76). In IBD patients, TNF disrupts the intestinal epithelial barrier, which makes the intestines vulnerable to infections, thus promoting inflammation (77). Mice overexpressing TNF develop chronic inflammation resembling IBD (77). However, TNF/lymphotoxin knockout or ectopic expression of mTNF delays the disease onset of EAE in mice (78, 79).

### TNFR1 in Autoimmune and Inflammatory Diseases

Activation of TNF/TNFR1 signaling predominantly promotes inflammation and tissue degeneration. Interaction of TNF with TNFR1 activates Teff cells and guides the migration of Teff cells to inflammatory sites (80); for example, CD4^+^ Teff cells are preferably accumulated in synovial joints in RA patients (81). Meanwhile, TNFR1 knockout prevents the development of arthritis and IBD in mice (82) and shortens the disease course of EAE and T1D in mice (78, 83), indicating a pro-inflammatory role of TNFR1 signaling. Furthermore, TNFR1 signaling likely impairs Treg functions *via* induction of the dephosphorylation of FoxP3 by protein phosphatase 1 in the inflamed synovium of RA, accompanied by increased numbers of Th17 and IFN^+^ CD4 T cells (84). Thus, TNF and TNFR1 have been used as therapeutic targets for the treatment of autoimmune and inflammatory diseases. An anti-TNFR1 nanobody protects against EAE development in mice (85), and sTNFR1 has been used as a natural inhibitor of TNFR1 signaling by binding and saturating TNF to repress its signaling (64).

### TNFR2 in Autoimmune and Inflammatory Diseases

In contrast to the pro-inflammatory effects of TNFR1, the TNF/TNFR2 interaction preferentially mediates immunosuppressive effects (86–89). In mice with dextran sulfate sodium-induced colitis, TNFR1 ablation exacerbated the severity of the disease, while TNFR2 deficiency led to the opposite results (90). TNFR2 knockout in EAE mice accelerated the disease progression accompanied by severe demyelination (78), suggesting a repressive role of TNFR2 in the disease development. Similarly, polymorphisms in TNFR2 have been found in various autoimmune diseases, which might lead to deregulation of TNF signaling *via* upregulation or shedding of TNFR2 (91).

TNFR2 has been identified as a marker for activated Tregs. TNFR2 and its ligands can activate and stabilize Tregs in an inflammatory environment (92–94). A subset of Tregs with high TNFR2 expression exhibits maximally suppressive activities in both mouse and human, which makes them the most desirable cells for the treatment of autoimmune and inflammatory diseases (95, 96). Furthermore, TNFR2 agonists have proved effective for the treatment of autoimmune disease (91, 97). Upon stimulation, TNFR2 is rapidly upregulated in Tregs, which are empowered to exert stronger immunosuppressive effects on Teff cells than non-stimulated Tregs (93).

However, stimulation of TNFR2 on Teff (e.g., Th1, Th17, and CD8^+^) cells promotes the cells to proliferate, secrete cytokines, and develop resistance to Treg-mediated suppression (95, 98–100). For example, the CD25^hi^/TNFR2^+^ Treg subset induced upon TCR stimulation allows the identification of maximal cytokine-producing effectors (101). These lines of evidence indicate the complex effects of TNFR2 on T cells, which help balance between Treg and Teff cells and partially explain the reasons for the controversial responses of some patients to TNFR2 agonists.

## Dual Effects of TNF on Autoimmune and Inflammatory Diseases in the Central Nervous System (CNS)

Although beneficial effects of TNF therapies have been observed in patients with RA, Crohn’s disease, SLE, and psoriasis, clinical trials on MS patients showed the opposite effects, with worsening of their symptoms (102). Adverse effects have also been found in trials on patients with optic neuritis, MS, and other demyelinating diseases following anti-TNF medications (103, 104). The adverse effects occurred in 0.05–0.2% of patients treated with three licensed anti-TNF agents. The opposing outcomes of TNF therapies may result from the dual effects of TNF on inflammation in the CNS.

Circulating TNF in the periphery can cross the blood–brain barrier (BBB) and enter the CNS. Infiltrating immune cells such as macrophages as well as activated microglia in the CNS can produce TNF (105). Generally, binding of TNF to TNFR1 predominantly mediates pro-inflammatory effects of TNF accompanied by activation of the target cells. In murine models of ischemia and EAE, TNFR1-ablation reduced neuronal loss and demyelination (105, 106). In addition, TNFR1 signaling activates microglia to promote neural inflammation due to increased production of pro-inflammatory factors including TNF, IL-1β, and IL-6 (107). TNF also induces apoptosis of human adult oligodendrocytes by causing mitochondrial dysfunction *via* TNFR1/JNK-3 signaling pathway and inhibits differentiation of oligodendrocyte progenitor cells (OPC) *via* AMPK activation and mitochondrial impairment (108, 109). These results indicate the adverse effects of TNFR1 signaling on multiple cell types in the CNS during the disease progression.

By contrast, upregulation of TNFR2 in OPC, microglia, and astrocytes promotes neuroprotection and remyelination, as observed in TNFR1-ablated mice with cerebral ischemia and EAE (105). TNFR2 ablation impairs OPC differentiation and causes dysfunction of oligodendrocytes (110). TNFR2 signaling promotes OPC differentiation and remyelination by inducing secretion of CXCL12 and leukemia inhibitory factor from astrocytes (111) and protects oligodendrocytes from oxidative stress-induced damage (112).

In addition, TNFR2 ablation in microglia in the CNS accelerates the onset of EAE, whereas disruption of TNFR2 in monocytes/macrophages suppresses the disease progression accompanied by reduction of T cell activation and infiltration, and attenuated demyelination (113), indicating that TNFR2 plays opposite roles even in microglia and macrophages during development of EAE. Activated microglia enhance the myelin debris clearance and remyelination, which is likely mediated by TNFR2 signaling (113, 114). These findings are instrumental for developing tissue- and receptor-specific medications to target TNF signaling in the treatment of different autoimmune and inflammatory diseases.

## TNF Regulation of MSC Efficacy on Autoimmune and Inflammatory Diseases

Interferon γ affects MSC efficacy in a dose-dependent manner. At low concentrations, it completely abolishes the therapeutic effect of MSCs on EAE, accompanied by increased secretion of the pro-inflammatory chemokine CCL2 and elevated expression of major histocompatibility complex molecules (115). At higher concentrations, IFNγ strengthens the MSC efficacy to reduce the severity of induced colitis in mice (27, 41). Similarly, TNF also dose dependently alters MSC functions. For example, osteogenic differentiation from murine ST2 MSCs is promoted by TNF at lower concentrations as indicated by elevated expression of the osteogenic genes *Runx2, Osx, OC*, and *ALP* but inhibited by TNF at higher concentrations, which depends on NFκB signaling (116). Compared with non-primed controls, TNF-primed MSCs have stronger immunomodulatory and tissue-repair capacity, evidenced by increased secretion of immunosuppressive molecules, such as PGE2, sTNFR, and TSG-6 (42, 117–123); chemokines, such as IL-8, CXCL5, and CXCL6 (124, 125); growth factors, such as HGF, IGF1, and VEGF (126–128); and increased tunneling nanotube (TNT) formation (129) through the TNFR1 or TNFR2 signaling pathway. The important effects of MSC through TNF signaling are listed in Table 1.

**Table 1:** Tumor necrosis factor (TNF) regulation of mesenchymal stem cell (MSC) efficacy on autoimmune and inflammatory diseases.

Disease	MSCs	Findings	Reference
Experimental autoimmune encephalomyelitis (mouse)	Mouse skin MSCs	Secrete soluble TNFR1 (sTNFR1) Inhibit differentiation of Th17 *via* sTNFR1-mediated TNF neutralization	(121)
	Human placental MSCs (TNF primed)	Express TSG-6 Attenuate disease severity	(122)

Systemic lupus erythematosus (SLE) (human)	BM-MSC (TNF primed) from SLE patients	Inhibit *in vitro* migration and *in vivo* homing capacity of BMSC Decrease hepatocyte growth factor production *via* the TNFR1/IKK-β pathway	(80)

Th1 cell induced pre-eclampsia (mouse)	Human decidual MSCs	Reverse abnormal TNF expression in uterine and splenic lymphocytes	(130)

Collagen-induced arthritis (CIA) (mouse)	Human BM-MSCs (expressing sTNFR2-Fc)	Secrete sTNFR2-Fc Decrease Th17 cell population Suppress osteoclastogenesis	(131)
	Mouse MSC line (TNF primed)	Secrete interleukin (IL)-6 Accentuate Th1 response No benefit on disease	(54)

Collagen II antibody-induced arthritis (mouse) or CIA (rat)	Human BM-MSCs (expressing sTNFR2-Fc)	Secrete sTNFR2-Fc Reduce joint inflammation	(132)

Ankylosing spondylitis (AS) (human)	Human BM-MSCs from AS patients (TNF primed)	Express TRAIL-R2 Induce MSC apoptosis *via* TRAIL-R2 and TNFR1 signal	(133)

Myocardial infarction (rat)	Rat BM-MSCs (overexpressing TNFR2)	Secrete sTNFR2 Attenuate expression of TNF, IL-1β, and IL-6	(134)
	Rat BM-MSCs (TNF primed)	Express TGFβ, FGF2, angiopoietin-2, and VEGF-1 Increase BM-MSC migration *in vitro*	(135)
	Mouse BM-MSCs	TNFR1 knockout Increases cardiac protection Decreases TNF, IL-1β, and IL-6 Increases VEGF in myocardium TNFR2 or TNFR1/2 knockout Reduces cardiac protection Increases TNF, IL-1β, and IL-6 Decreases VEGF in myocardium	(136)

Myocardial infarction (mouse)	Human BM-MSCs (TNF primed)	Express TSG-6 Decrease inflammatory responses Reduce infarct size Improve cardiac function	(123)

Myocardial ischemia–reperfusion injury (rat)	Mouse BM-MSCs	TNFR1 knockout increases the cardioprotective effect in male but not in female MSCs	(137)
	Mouse BM-MSCs	TNFR1 (but not TNFR2 or TNFR1/2) knockout MSCs increase the cardioprotective effect	(138)

Anthracycline-induced cardiomyopathy (mouse)	Human induced pluripotent stem cell-MSCs/human BM-MSCs (TNF primed)	Express MCP-1, IL-6, IL-8, and VEGF Form tunneling nanotubes for mitochondria transfer *via* TNF/NFκB/TNFαIP2 signal	(129)

Inflammatory dilative cardiomyopathy or LPS-induced acute lung injury (mouse)	Mouse BM-MSCs	Secrete sTNFR1 to neutralize TNF and LTα Suppress NFκB pathway in cardiomyocytes	(120)

Ischemic hindlimb (mouse)	Human ASCs (TNF primed)	Secrete IL-6 and IL-8 Promote angiogenesis, chemotactic migration of human cord blood-derived endothelial progenitor cell	(139)

Sepsis (mouse)	Mouse BM-MSCs (TNF primed)	Express COX2 to synthesize PGE2, which increases *IL10* expression in macrophages *via* TNF/TNFR1 signaling	(118)

LPS intoxication (systemic inflammation) (rat)	Human BM-MSCs (LPS intoxication serum primed)	Promote sTNFR1 secretion *via* NF-κB signaling Decrease TNF, interferon γ, and IL-6 Decrease infiltration of macrophages and neutrophils	(119)

Pig islet xenotransplantation in streptozotocin-induced diabetes model (humanized mouse)	Human ASCs (sTNFR1-Fc)	Improve survival of porcine islets Reverse hyperglycemia	(140)

Cutaneous wound (rat)	Human ASCs (TNF primed)	Express IL-6 and IL-8 Enhance macrophage infiltration Enhance cell proliferation and angiogenesis	(141)

Experimental allergic conjunctivitis (mouse)	Human BM-MSCs (TNF primed)	Express COX-2 to synthesize PGE2 Decrease IgE production and histamine release Decrease conjunctival vascular hyperpermeability	(117)

### TNFR1-Mediated Regulation of MSC Efficacy

Generally, TNFR1-mediated signaling reduces the MSC efficacy. For example, BM-MSCs derived from mice with TNFR1 knockout caused greater recovery of myocardial functions in a rat model of acute ischemia than wild-type MSCs, which was associated with increased production of VEGF and decreased production of the pro-inflammatory factors TNF, IL-1β, IL-6, etc., in the myocardium (136, 138). Interestingly, another study found that TNFR1 knockout only increased the cardioprotective effect of male, but not female, MSCs in a murine ischemic injury model (137), indicating that the effect of TNFR1 signaling is gender dependent.

TNFR1 signaling reduces MSC efficacy by inhibiting the production of immunosuppressive molecules and growth factors. For example, TNF-priming reversed the immunosuppressive effect of mouse MSCs on T cell proliferation, accompanied by increased secretion of the pro-inflammatory cytokine IL-6 and failure of the MSCs in the treatment of murine CIA (54). In addition, ablation of TNFR1 remarkably increased TNF-stimulated HGF production from human BM-MSCs (142), indicating the inhibitory effect of TNFR1 signaling in HGF production. Similar effects have been observed on MSCs derived from patients with autoimmune diseases. For instance, it has been shown that TNF treatment decreased the HGF production by BM-MSCs derived from SLE patients *via* the TNFR1/IKK-β pathway (80) and induced apoptosis in BM-MSCs from ankylosing spondylitis patients *via* TNFR1-mediated upregulation of *TRAIL-R2* (133).

Interestingly, in some scenarios, TNFR1 signaling can enhance MSC efficacy by inducing production of immunomodulatory molecules. For example, TNFR1 knockdown in mouse skin-derived MSCs abrogated their therapeutic effects on EAE accompanied by reduced inhibition on the polarization of Th17 cells (121), which might be partially explained by the loss of beneficial effects of sTNFR1 produced by MSC under the inflammatory situation. In addition, in dilative cardiomyopathy, acute lung injury, and LPS-induced intoxication, both murine and human BM-MSCs primed by TNF or inflammatory serum secreted more sTNFR1 than the non-primed controls, which promotes disease recovery (119, 120). In addition, human adipose-derived MSCs engineered to express sTNFR1-Fc improved the survival of porcine islets and reversed the hyperglycemia in a mouse model of streptozotocin-induced diabetes (140). sTNFR1 may act by neutralizing circulating TNF and activating mTNF-mediated reverse signaling in immune cells during diseases progression.

TNFR1 signaling can also increase PGE2 secretion by inducing *COX2* expression in mouse or human BM-MSCs, which in turn reprograms host macrophages to increase IL-10 production thus inhibiting inflammation in a mouse sepsis model and experimental allergic conjunctivitis (117, 118). In addition, it has been shown that other immunosuppressive molecules, growth factors, and chemokines such as TSG-6, TGFβ, and IL-8 were produced by TNF-primed MSCs to attenuate the symptoms in diseases including EAE, myocardial infarction, ischemic hind limb, and cutaneous wound probably *via* TNFR1 signaling pathway (122, 135, 139, 141). TNF can also induce TNT formation between iPSC-derived MSC and cardiomyocytes for mitochondria transfer to attenuate the damage in mouse anthracycline-induced cardiomyopathy, which is regulated by TNF/NFκB/TNF-IP2 signaling pathway (129). Thus, TNFR1 signaling can exert dual effects on MSC-based therapy in autoimmune and inflammatory diseases, depending on the type and stage of the diseases.

### TNFR2-Mediated Regulation of MSC Efficacy

In contrast to the dual effects of TNFR1, TNFR2-mediated signaling enhances MSC efficacy in general. For example, compared with wild-type controls, both male and female murine BM-MSCs with TNFR2 knockout showed less or no myocardial functional recovery in a rat model of acute ischemia accompanied by increased production of pro-inflammatory factors and a reduced level of VEGF in the myocardium (136, 138). These results are consistent with the *in vitro* observations that production of VEGF, IGF-1, and HGF by TNF-primed human BM-MSCs is mediated through the TNFR2 signaling (126–128). Consistently, TNFR2 knockout reduced the secretion of VEGF and IGF-1 by TNF-primed BM-MSCs, but this only happened on BM-MSC from female mice. By contrast, secretion of these growth factors increased in TNF-primed TNFR2^−/−^ BM-MSCs from male mice (143, 144), and TNFR2^−/−^ BM-MSCs from male mice failed to promote myocardial functional recovery (136, 138). The opposite outcomes implicate that the effects of TNFR2 signaling, like TNFR1 signaling, on MSC functions are also gender dependent. In support of this, the male sex hormone testosterone has been reported to exert deleterious effect on myocardial recovery in a rat model (145, 146).

Furthermore, overexpression of *sTNFR2* or *TNFR2* in human or rat BM-MSCs enhanced their therapeutic effects in mice and rats with RA (131, 132) and rats with cardiac ischemia (134, 147), which was associated with reduced TNF level and attenuated expression of *IL1β* and *IL6*. Macrophages are a major cell type that secretes TNF. Treating activated macrophages with culture supernatant of human sTNFR2-expressing MSCs reduced osteoclast formation *in vitro* (131). Similar to sTNFR1, sTNFR2 may also execute cytoprotective effect *via* neutralization of circulating TNF or induction of mTNF-mediated reverse signaling in immune cells.

The expression of *TNFR2* is highly upregulated in oligodendrocytes, microglia, astrocytes, and several subsets of neurons in neurological diseases (105, 148). TNFR2 on astrocytes mediates beneficial activities to protect oligodendrocytes in co-culture (111). Upregulated TNFR2 on activated microglia promotes the clearance of myelin debris and remyelination (149). In addition, MSCs that infiltrate into the CNS can exert immunomodulatory effects by regulating the local microglia and astrocytes as well as infiltrating immune cells, e.g., suppressing the functions of Teff cells and macrophages and promoting the proliferation of Tregs (150). Moreover, TNF in inflamed CNS induces MSC to secrete immunomodulatory factors and neural tropic factors such as BDNF and HGF (151), which exert pleiotropic effects to attenuate the brain inflammation, reduce brain damage, and promote neural regeneration.

## TNF Signaling Interacting with MSCs on Tregs

Regulatory T cells play a central role in the maintenance of the immune balance to tolerate self-antigens and prevent autoimmunity (152). In general, they refer to CD4^+^/FOXP3^+^ T cells, including two major subtypes: natural Treg (nTreg) cells and induced adaptive Treg (iTreg) cells. nTreg cells are generated and selected in the thymus and then migrate to peripheral tissues (153), while iTreg cells acquire CD25 (IL-2Rα) expression outside of the thymus and are typically induced by inflammation and during disease processes, such as autoimmunity and cancer (152). T cell receptor stimulation and the cytokines TGFβ and IL-2 are required for iTreg cell generation *in vitro* and *in vivo* (95, 154, 155). In contrast to the pro-inflammatory effects of TNF/TNFR1 signaling (156), TNF/TNFR2 signaling preferentially activates, stabilizes, and expands Tregs to mediate their immunosuppressive effects and contribute to the treatment of autoimmune disease (86–89). TNFR2 is an expression marker relevant to Treg functions. TNFR2 agonists have been shown to be effective for the treatment of autoimmune and inflammatory diseases (91, 97).

Mesenchymal stem cells regulate both innate and adaptive immune systems partially by promoting the generation of Tregs (29–31). In the presence of high levels of inflammatory cytokines, e.g., TNF and IFNγ, MSCs produce various soluble factors, such as IDO, TGFβ, PGE2, and IGF, to inhibit Teff cells and increase the expression of *FOXP3, CTLA4*, and *GITR* in Tregs to enhance their immunosuppressive effects (53). Cell-to-cell contact also mediates the induction of Tregs by cytokine-primed MSCs (53). Overexpression of inducible co-stimulator ligands in MSCs promotes the induction of functional Tregs (157).

In addition, MSCs also modulate antigen-presenting cells, such as DCs and macrophages, by converting them to anti-inflammatory phenotypes (M2), which then promote Treg expansion and suppress Teff cell functions (30). Recently, Miyagawa et al. reported that MSCs control Treg proliferation by releasing IGFBP4, an inhibitor of IGF (53). Moreover, some studies have shown that low levels of IFNγ and TNF or long-term exposure to these cytokines converts MSC from an immunosuppressive to pro-inflammatory status (53–55). Thus, these pro-inflammatory cytokines can modify MSC effects on Tregs, altering their efficacy on autoimmune and inflammatory diseases.

## Strategy and Perspective

Mesenchymal stem cells have demonstrated immunosuppressive effects against various autoimmune and inflammatory diseases. However, the efficacy of MSC on many of the diseases remains controversial, which can be attributed to many reasons. The first is the challenge MSCs encounter when adapting to a new microenvironment following delivery into the body. They have to first survive in the new and often harsh conditions, during which the MSC effects can be reduced or even lost. Thus, improvement of the MSC efficacy should focus on achieving high delivery efficiency, long-term retention, and specific modification to target different inflammatory diseases.

Genetically modified MSCs can gain remarkably enhanced therapeutic capability, in which MSCs serve as a carrier to deliver cytokines or verified biological drugs for target-oriented therapies. For example, compared with unmodified MSCs, MSCs transduced with TGFβ suppressed CIA in a mouse model (158). MSCs expressing IL-12p40 alleviate murine colitis more effectively than a wild-type control (159). Overexpressing IL-10 in MSCs suppressed the development of graft-versus-host disease (160), and MSCs overexpressing TNFR2 treat CIA in mouse more effectively than controls (131). MSCs can also be engineered to release abundant amounts of sTNFR1 to neutralize TNF in the circulation (121, 140). In addition, since MSCs promote activation and proliferation of Tregs, combined therapy of MSCs and Tregs further enhances the number and functions of Tregs and achieves much stronger efficacy than each alone, which has been observed in GVHD (161, 162) and ischemic myocardium (163).

## Concluding Remarks

**Figure 1:**
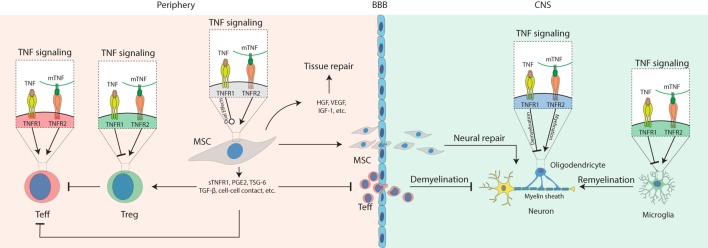
Schematic diagram for the role of tumor necrosis factor (TNF) signaling in mesenchymal stem cell (MSC)-based therapy on autoimmune and inflammatory diseases. Under inflammatory conditions, TNF binds TNFR1 to activate T effector (Teff) cells while impairing regulatory T cells (Tregs); mTNF mostly binds TNFR2 to activate Teff cells and also activate Tregs to mediate their immunosuppressive effects in the periphery. In the central nervous system (CNS), TNFR1 signaling induces cytotoxic effects on oligodendrocytes resulting in neural demyelination and activates microglia to produce pro-inflammatory molecules such as TNF. TNFR2 signaling protects the survival of oligodendrocyte and microglia and promotes myelin clearance and remyelination mediated by microglia. In addition, TNFR1 or -R2 signaling can enhance the immunosuppressive effects of MSCs to alleviate autoimmune and inflammatory diseases. Compared to non-primed controls, TNF-primed MSC produce more soluble TNFR1 (sTNFR1), PGE2, TSG-6, and TGF-β, enhance Treg functions, neutralize TNF *via* sTNFR, prevent Teff cell infiltration into the CNS, release growth factors such as hepatocyte growth factor (HGF), insulin-like growth factor-1 (IGF-1), and VEGF to promote tissue or neural repair, infiltrate the CNS to mediate neural protection by regulating oligodendrocytes and microglia, suppressing Teff cells that have infiltrated the CNS.

In this review, we describe the progress in research on how TNF signaling interacts with MSCs in the treatment of autoimmune and inflammatory diseases (Figure 1). At appropriate concentrations and timing, TNF promotes secretion of immunosuppressive molecules from MSCs, which inhibit Teff cells and activate Tregs. In the periphery, TNFR2 signaling also stimulates Tregs; thus, it may synergize with MSCs to repress inflammation. In the CNS, TNFR2 signaling protects the survival of astrocytes, OPC, microglia, and neurons. Activated MSCs secrete immunosuppressive molecules to inhibit inflammation and neurotropic molecules to protect neural cells and promote remyelination. Some of the TNF functions mediated by either TNFR1 or -R2 in MSCs can vary in different genders. Together, these findings suggest that TNF signaling plays a pivotal role in MSC-based therapy of autoimmune disease, which is highly dependent on the context, timing, concentration, gender, etc.

Despite these interesting findings, many more questions remain to be addressed than have been solved. For example, how do transplanted MSCs respond to TNF, function in the periphery and infiltrate the inflamed CNS in patients. Why does gender affect TNF functions? Would genetic variations among different individuals affect TNF functions? Can inflammatory factors also epigenetically modify and alter the expression of the genes involved in TNF signaling? Future studies are needed to address these and many new challenging questions. Continuous progress in this field will most likely lead to the identification of new targets for more precise and effective therapies of autoimmune and inflammatory diseases.

## Author Contributions

LY, DZ, and R-HX conceived, designed, and wrote the manuscript. R-HX gave the final approval of the manuscript.

## Conflict of Interest Statement

RH-X is a founder of ImStem Biotechnology, Inc., a stem cell company. He declares competing financial interests. No financial conflicts of interest exist for any of the authors.
